# Cathepsin E Deficiency Ameliorates Graft-versus-Host Disease and Modifies Dendritic Cell Motility

**DOI:** 10.3389/fimmu.2017.00203

**Published:** 2017-03-01

**Authors:** Jörg Mengwasser, Liane Babes, Steffen Cordes, Sarah Mertlitz, Katarina Riesner, Yu Shi, Aleixandria McGearey, Martina Kalupa, Thomas Reinheckel, Olaf Penack

**Affiliations:** ^1^Medical Department, Division of Hematology, Oncology and Tumor Immunology, Charité University Medicine, Berlin, Germany; ^2^Faculty of Medicine, BIOSS Centre for Biological Signalling Studies, Institute of Molecular Medicine and Cell Research, Albert-Ludwigs-University Freiburg, Freiburg, Germany

**Keywords:** GVHD, cathepsin E, dendritic cells, HSCT, motility

## Abstract

Microbial products influence immunity after allogeneic hematopoietic stem cell transplantation (allo-SCT). In this context, the role of cathepsin E (Ctse), an aspartate protease known to cleave bacterial peptides for antigen presentation in dendritic cells (DCs), has not been studied. During experimental acute graft-versus-host disease (GVHD), we found infiltration by Ctse-positive immune cells leading to higher Ctse RNA- and protein levels in target organs. In Ctse-deficient allo-SCT recipients, we found ameliorated GVHD, improved survival, and lower numbers of tissue-infiltrating DCs. Donor T cell proliferation was not different in Ctse-deficient vs. wild-type allo-SCT recipients in MHC-matched and MHC-mismatched models. Furthermore, Ctse-deficient DCs had an intact ability to induce allogeneic T cell proliferation, suggesting that its role in antigen presentation may not be the main mechanism how Ctse impacts GVHD. We found that Ctse deficiency significantly decreases DC motility *in vivo*, reduces adhesion to extracellular matrix (ECM), and diminishes invasion through ECM. We conclude that Ctse has a previously unrecognized role in regulating DC motility that possibly contributes to reduced DC counts and ameliorated inflammation in GVHD target organs of Ctse-deficient allo-SCT recipients. However, our data do not provide definite proof that the observed effect of Ctse^−/−^ deficiency is exclusively mediated by DCs. A contribution of Ctse^−/−^-mediated functions in other recipient cell types, e.g., macrophages, cannot be excluded.

## Introduction

The biggest threat for patients receiving a potentially life-saving allogeneic hematopoietic stem cell transplantation (allo-SCT) is the development of severe graft-versus-host disease (GVHD). The consequences of GVHD are organ damages, mainly in liver, intestines, and skin, leading to substantial morbidity and mortality. Recent research identified important mechanisms regarding the induction of GVHD through the interplay of microbial molecules and innate immunity, thus opening a new area for future therapeutic approaches (1–5).

Cathepsin E (Ctse) is an aspartic endopeptidase belonging to the cathepsin family of proteases. Ctse is mainly expressed and active in antigen-presenting cells of the immune system, such as dendritic cells (DCs) and macrophages (6–8). The main known common function of cathepsin proteases is to liberate peptide epitopes for antigen presentation (9, 10). Ctse was shown to be involved in antigen presentation of bacterial peptides in DCs, and it may also be important for the presentation of allogeneic peptides on MHC class II molecules (11–13). Recent studies demonstrated an increase in Ctse protein expression or enzymatic activity in patients suffering from Parkinson disease (14) or *Helicobacter pylori* infections (15). Ctse overexpression has been associated with several types of cancer (16–21) and has been used as a predictive biomarker in patients with malignant diseases (22–24). The role of Ctse for the pathophysiology of GVHD has not been studied experimentally.

To be able to analyze the impact of Ctse *in vivo*, we have previously created *Ctse*–null mice, which have a normal phenotype (25). OVA-antigen processing and presentation by DCs showed no difference in *Ctse^+/+^* and *Ctse*^−/−^ mice *in vivo* under non-inflammatory conditions. In experimental models of allergic airway inflammation, we found that Ctse deletion results in a reduced inflammatory response, marked by impaired lymphocyte infiltration into lungs in comparison to wild-type (WT) littermates (25). In the current study, we examined the role of Ctse during GVHD after allo-SCT and its influence on DC function.

## Materials (or Subjects) and Methods

### Mice

Female C57BL/6 (H2^b^), 129S2/SvPasCrl (H2^b^), and B6D2F1 (H2*^b/d^*^)^, female and male LP/J (H2^b^) mice (10–12 weeks old) were purchased from Charles River Laboratories (Sulzfeld, Germany) and Jackson Laboratory (Bar Harbor, ME, USA). Female *Ctse*^−/−^ and *Ctse^+/+^* mice (25) were bred by and obtained from the central animal unit of the Charité University Medicine. Mice had access to food and water *ad libitum*. The Regional Ethics Committee for Animal Research (State Office of Health and Social Affairs Berlin) approved all animal experiments. Number: G0118/13. Dr. Penack and Dr. Mengwasser. Title: Ctse in allo-HSCT.

### Conditioning Regimen

Busulfan (Sigma-Aldrich, St. Louis, MO, USA) was dissolved in DMSO (40 mg/ml) and cyclophosphamide monohydrate (Sigma-Aldrich) in sterile water (10 mg/ml). Female C57BL/6 received IP doses of busulfan (20 mg/kg/day) for 4 days, followed by cyclophosphamide (100 mg/kg/day) for 2 days. Days -2 and -1 prior to HSCT were resting days.

### Bone Marrow (BM) Transplantation (Stem Cell Transplantation, SCT)

We have recently described a chemotherapy-based MHC-matched miHA-mismatched murine GVHD model, which may better resemble human GVHD as compared with traditional radiation-based MHC-mismatched models (26). In this model, C57BL/6 mice are used as allo-SCT recipients. Therefore, we were able to use this specific model for all studies investigating Ctse^−/−^ on the recipient side. For investigation of Ctse^−/−^ on the donor side, we had to use an alternative model, because no reliable chemotherapy-based miHA-mismatched models exist with C57BL/6 mice on the donor side. We opted for the next best situation and used a haploidentical model for testing of Ctse^−/−^ on the donor side. Recipient C57BL/6 mice were injected i.v. with 1.5 × 10^7^ BM cells and 2 × 10^6^ splenic T cells from LP/J (allogeneic) and C57BL/6 (syngeneic) donor mice on day 0. BM from the tibia and femur was flushed with isolation buffer (PBS/2% FCS/1 mM EDTA) and single-cell suspension was prepared by gently passing through a 23G needle and over a 70 μm cell strainer (BD Biosciences, San Jose, CA, USA). Splenocytes were passed through a 40 μm cell strainer. Erythrocytes were lysed with ammonium chloride buffer (Sigma-Aldrich). Splenic T cell was obtained using the mouse Pan T Cell Isolation Kit II (Miltenyi Biotec, Bergisch Gladbach, Germany) according to manufacturer’s instructions. T cell purity was analyzed by CD3 staining and FACS analysis.

### GVHD Monitoring

Mice were individually scored twice a week for five clinical parameters (posture, activity, fur, skin, and weight loss) on a scale from 0 to 2. Clinical GVHD score was assessed by summation of these parameters. Survival was monitored daily.

### Tumor Experiments

C57BL/6 mice received 1 × 10^6^ EL4 (C57BL/6) T cell lymphoma tumor cells stably expressing firefly luciferase intravenously. Tumor growth was measured by bioluminescence imaging. 3 mg/mouse d-luciferin (Xenogen, Alameda, CA, USA) was injected i.p., mice were anesthetized and placed in an IVIS 200 system. Average radiance (p/s/cm^2^/sr) was determined using the Living Image 3.1 software (PerkinElmer, Waltham, MA, USA).

### FACS Analysis

Peripheral blood was lysed for 10 min with ammonium chloride. BM, spleen, lymph node, and thymus were prepared consistent to the BMT Protocol. Single-cell suspensions were washed two times, collected in MACS buffer (PBS/0.5 mM EDTA/0.5% BSA), and stained for 20 min at 4°C with rat anti-mouse antibodies from BD Biosciences. Antibodies used are anti-Ly9.1-PE (30C7), APC-conjugated anti-CD8a (53-6.7), PerCP-Cy5.5-conjugated anti-CD45R/B220 (RA3-6B2), FITC-conjugated anti-H2kb (AF6-88.5), PE-Cy7-conjugated anti-CD4 (RM4-5), anti-CD11c (HL3), APC-Cy7-conjugated anti-CD3e (145-2C11), and PerCPCy5.5-conjugated anti-mouse CD86 (GL-1). Samples were analyzed using a BD FACSCanto II (BD Biosciences) and FlowJo 7.6.5 Software (Tree Star Inc., Ashland, OR, USA).

### Histology

Tissue samples were harvested and cryo-embedded in Tissue-Tek optimum cutting temperature compound (Sakura Finetek, Alphen aan den Rijn, Netherlands). Cryosections (5 μm) were acetone fixed at −20°C. Histopathological score was assessed on H&E-stained sections after Lerner criteria (27) and grade of liver inflammation [slight (1) or marked (2) portal/venous and marked lobular (3) inflammation]. Sections for analysis of lymphocyte infiltration were blocked by PBS/3% BSA/5% FCS and stained over night at 4°C with primary rat anti-mouse antibodies against, CD11c (HL3), CD4 (H129.19), and CD8a (53-6.7) from BD Biosciences. Binding was visualized with secondary donkey anti-rat antibody conjugated with Alexa Fluor 488 or Cy3 (Life Technologies) and nuclear counterstain was performed using 4,6-diamidino-2-phenylindole (Sigma-Aldrich). Five sections per sample were investigated with a Motic BA410 epifluorescence microscope (Motic, Hong Kong) and quantification was assessed by calculating CD11c^+^, CD4^+^, or CD8^+^ area against total liver parenchyma or colon mucosal area with a predetermined threshold using Fiji Software (http://fiji.sc/Fiji).

### Image Acquisition

Fluorescence and bright-field images were taken using a Motic BA410 microscope with a Moticam Pro 285B and the Motic Images Plus 2.0 software. The used objectives were Plan Fluar 10×/0.30, 20×/0.50, and 40×/0.75. Adobe Photoshop CS6 was used for image processing.

### Anti-Mouse Ctse Polyclonal Antibody Generation

The following peptide sequence of mouse Ctse: SLITGPPDKIKQLQE, which represents amino acids 286–300 of the mouse Ctse protein was chosen for immunization. Immunization of rabbits was done with the specific mouse Ctse peptide bound to a protein carrier (KLH) for 6 months, boosting once per month. Rabbit serum was isolated from positively tested animals, and the IgG fraction was purified.

### DC Generation

Dendritic cells (Ctse*^+/+^* and Ctse^−/−^) were generated from fresh isolated BM cells grew in CellGro DC medium (CellGenix) containing 10% FCS (Pan Biotech), IL-4 (10 ng/ml) (R&D), and GM-CSF (10 ng/ml) (GIBCO). Medium was changed on days 2 and 4. On the seventh day, cells were analyzed by flow cytometry using the markers CD11c, F4/80, CD14, MHCII, CD11b, CD80, CD86, CD45, CD3, and CD19.

### FITC Sensitization Experiment

Mice (Ctse*^+/+^*, Ctse^+/−^, and Ctse^−/−^) were anesthetized with Ketamin/Rompun (1:1). FITC (SIGMA) (10 mg/ml) was dissolved in equal volumes of acetone/dibutylphthalate (1:1) and applied with a pipet tip to the ear skin and the dorsal skin of Ctse*^+/+^* and Ctse^−/−^ mice. Lymph nodes (inguinal, mandibulares, retropharyngeus lateralis, axillaris, accesorius, and brachial) were removed 24 h later. Cell suspensions of total lymph nodes were prepared by straining the nodes through a nylon mesh filter (70 μm) and analyzed by flow cytometry. CD11c was used as DC marker. A total of 50,000 cells were counted and the relative migration of DCs was calculated by dividing the CD11c^+^ DCs through the sum of FITC^+^CD11c^+^ DCs and CD11c^+^ DCs.

### Adhesion Assay

The adhesion of DCs (Ctse*^+/+^* and Ctse^−/−^) was measured with the xCELLigence system (Roche) using E-plates (16 wells). Cells adhere to the bottom of the wells and lead to a change in the electrical impedance, which is measured by gold microelectrodes. The more cells adhere the higher increases the electrical impedance, which is displayed as Cell Index. The E-plates were coated with different substrates: collagen I 1.73 mg/ml (BD Biosciences), fibronectin 10 μg/ml (Sigma), and matrigel 9 mg/ml (Cultrex). A total of 50,000 DCs were activated with LPS in the well (4 μg/ml). Cell adhesion was measured within 2 h.

### Invasion Assay

Invasion of Ctse*^+/+^* and Ctse^−/−^ DCs was investigated with xCELLigence system (Roche). Microporous membrane (3 μm) in CIM-plates was either coated with a 1-mm thick layer of collagen I 1.73 mg/ml (BD Biosciences) or matrigel 9 mg/ml (Cultrex). The gold microelectrodes are attached below the membranes. A total of 80,000 DCs (Ctse*^+/+^* and Ctse^−/−^) were seeded on coated membranes in the upper compartment in the presence of LPS (4 μg/ml). In the lower compartment, medium with chemokines CCL21 (500 ng/ml) and CCL19 (500 ng/ml), both acting as CCR7 ligands, was present as a chemoattractant.

### *In Vivo* T Cell Proliferation Assay

C57BL/6 Ctse^−/−^ or C57BL/6 WT littermates were conditioned with chemotherapy as described above. CD3^+^ lymphocytes were isolated from spleens of either Balb/C or 129/J mice using mouse Pan T cell isolation Kit II (Miltenyi Biotec, Bergisch Gladbach, Germany) according to manufacturer’s instructions. Cells were loaded with CFSE (carboxyfluorescein diacetate, succinimidyl ester) at a final concentration of 2.5 μmol for 8 min at 37°C in 10% FCS/PBS. 5 × 10^6^ CFSE-loaded CD3^+^ cells were injected i.v. into the tail vein at day 0. Ninety-six hours later, mice were sacrificed, spleens and lymph nodes were taken and cells were isolated. After staining with the appropriate donor marker (H2kd for Balb/C and Ly9 for 129/J), cell samples were analyzed using a BD FACSCanto II (BD Biosciences) and FlowJo 7.6.5 Software (Tree Star Inc., Ashland, OR, USA).

### Mixed Leukocyte Reaction (MLR)

Dendritic cells were isolated from spleen of C57BL/6 Ctse^−/−^ and WT mice using a CD11c^+^ isolation kit and splenic T cells from LP/J mice were obtained using the mouse Pan T cell isolation Kit II (Miltenyi Biotec, Bergisch Gladbach, Germany) according to the manufacturer’s instructions. DCs were treated with 100 ng/ml LPS or left untreated for 4 h. T-cells were loaded with CFSE (5-(and-6)-carboxyfluorescein diacetate, succinimidyl ester, Thermo Fisher). 2.5 × 10^4^ DCs (activators) and 2.5 × 10^5^ T-cell (responders) were put together for 96 h in an incubator at 37°C and 5% CO_2_. FACS analysis was done measuring total cell counts positive for CFSE and CD3. Proliferating cells are determined as cells, showing less CFSE load compared to control samples with only CFSE-loaded T-cells.

### Real-time PCR

RNA and cDNA were obtained using the RNeasy Mini Kit (QIAGEN, Venlo, Netherlands) and the QuantiTect Reverse Transcription Kit (QIAGEN) after manufacturer’s instructions. Real-time PCR amplification reaction (50°C for 2 min, 95°C for 10 min, 49 cycles of 95°C for 10 s, 60°C for 1 min) was performed on DNA Engine Opticon (BioRad, Hercules, CA, USA) using the TaqMan Gene Expression Master Mix (Life Technologies) and primers and probes (BioTez GmbH, Berlin, Germany) designed with the Primer Express 1.5 software (Life Technologies). Data were analyzed with the Opticon Monitor 3.1 analysis software (BioRad) and the comparative CT method (ΔΔCT method).

### Statistics

Survival data were analyzed using the Kaplan–Meier method and compared with the Mantel–Cox log-rank test. For statistical analysis of all other data, rank sum tests were used, unless indicated otherwise. Values are presented as mean ± SEM. Values of *p* ≤ 0.05 were considered statistically significant. All statistical analyses were performed using GraphPad Prism software (GraphPad Software Inc., La Jolla, CA, USA).

## Results

### Ctse Is Overexpressed in Target Organs during Acute GVHD

**Figure 1:**
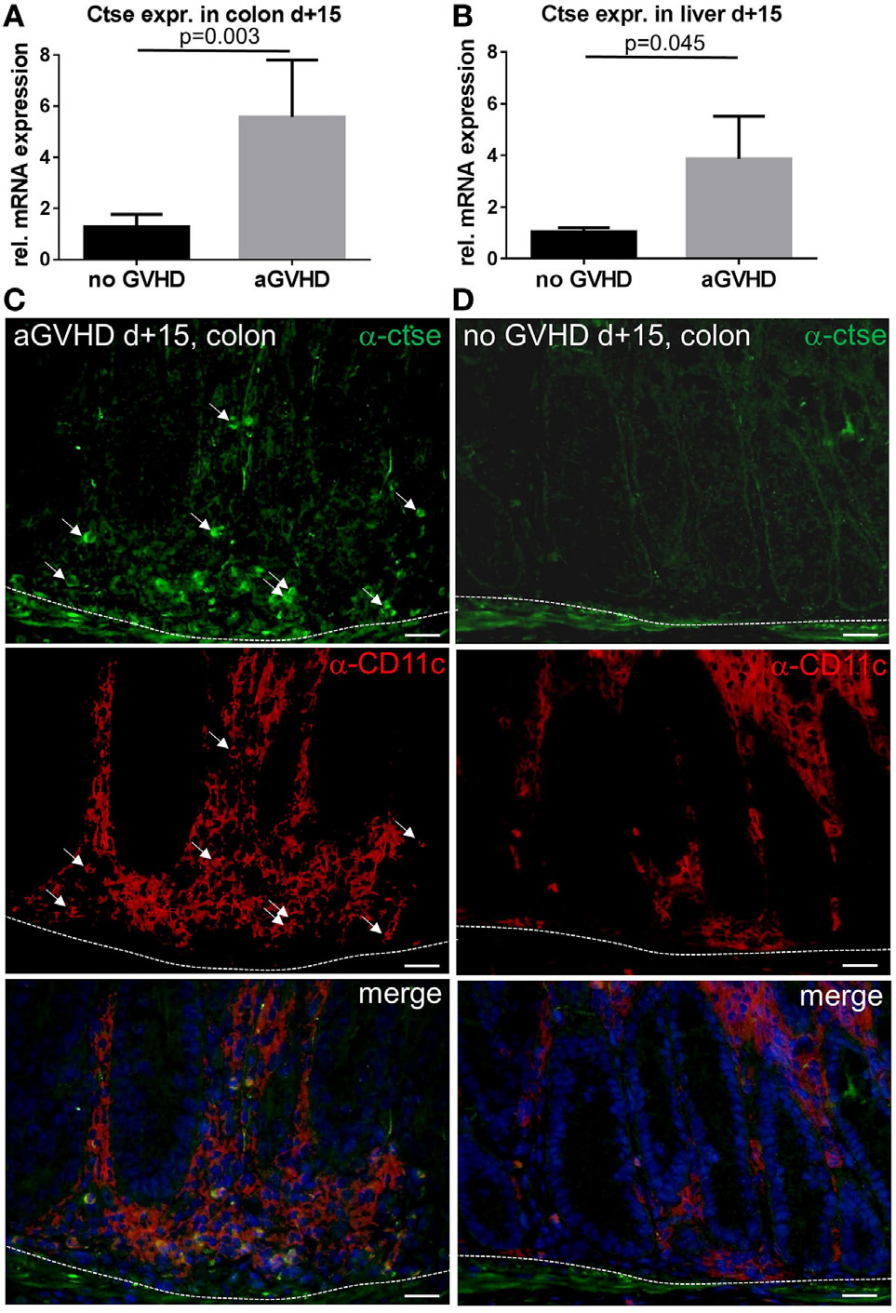
**Cathepsin E (Ctse) expression is increased in target organs during graft-versus-host disease (GVHD)**. **(A,B)** Ctse mRNA expression assessed by qPCR in colon and liver during acute GVHD [aGVHD, allogeneic stem cell transplantation; allogeneic hematopoietic stem cell transplantation (allo-SCT)], and syngeneic transplanted control animals (no GVHD, syn-SCT) at day +15 after allo-SCT in the LP/J→C57BL/6 model. Ctse expression is shown relative to the no GVHD control group. *n* = 5 per group **(C,D)** Ctse protein expression is elevated in immune cell infiltrates in the colon during acute GVHD (aGVHD) compared to syn-SCT recipients without GVHD (no GVHD). Ctse expression is shown in green, CD11c is shown in red, nuclear counterstain with DAPI is shown in blue, Ctse-expressing cells are highlighted with white arrows, same arrow positions are shown in the CD11c staining, illustrating that Ctse-positive cells are also CD11c^+^. Dotted line marks the border of the lamina propria to the lamina muscularis mucosae. Fluorescence images were taken using a Motic BA410 microscope with a Moticam Pro 285B and the Motic Images Plus 2.0 software. The used objective was a Plan Fluar 40×/0.75. Error bars indicate mean ± SEM, *p*-values in **(A,B)** were calculated using a double-sided Student’s *t*-test. Bar = 50 μm.

First, we analyzed the expression profile of Ctse in target organs during acute GVHD in the murine MHC-matched, miHA-mismatched LP/J→C57BL/6 (B6) allo-SCT model (26). Using qPCR and the comparative ΔΔCT method, we found a significant increase in Ctse mRNA levels in allo-SCT recipients with GVHD (aGVHD) relative to syngeneic (no GVHD) SCT recipients without GVHD in colon (mean: 5.58-fold increase in aGVHD vs. no GVHD, *p* = 0.003) and liver (mean: 3.87-fold increase in aGVHD vs. no GVHD, *p* = 0.045) at day +15 after allo-SCT (Figures 1A,B). Of note, the mean absolute mRNA expression level of Ctse in colon was more than 10 times higher as compared with liver (data not shown). In our hands, no commercially available antibody against Ctse worked in histology. Therefore, we produced a polyclonal rabbit anti-murine Ctse antibody choosing a different mouse Ctse peptide epitope for immunization as described in the Section “Materials (or subjects) and Methods.” We checked the antibody for its specificity using Ctse KO and WT tissues in immunofluorescence, obtaining no staining in Ctse KO tissues. Using this antibody in immunofluorescence analysis of cryosections, we confirmed that mRNA expression correlated with protein levels. In liver, protein expression was nearly undetectable, reflecting the results of the low mRNA expression level. Ctse protein expression was specifically increased in CD11c^+^ immune cell infiltrates in the colon in allo-SCT recipients (Figure 1C) during acute GVHD, but not in syn-SCT recipients (Figure 1D).

### Ctse Deficiency in Donor BM Cells and Donor T Cells Does Not Affect the Development of GVHD

**Figure 2:**
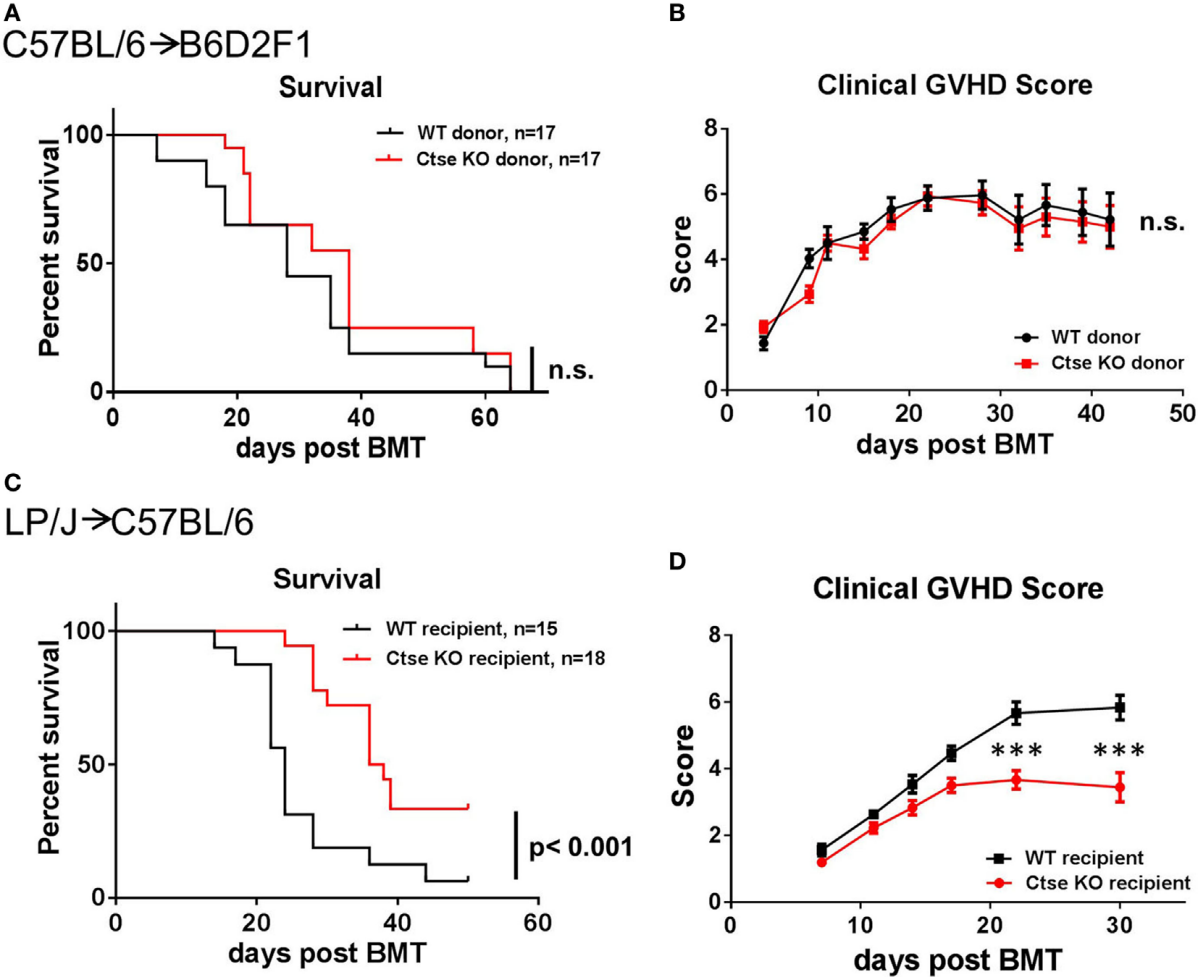
**Lethal graft-versus-host disease (GVHD) is reduced in cathepsin E (Ctse)-deficient allogeneic hematopoietic stem cell transplantation recipients**. **(A,B)** No significant differences were observed when wild-type (WT) and Ctse^−/−^ (Ctse KO) C57Bl/6 were used as donors in the C57BL/6→B6D2F1 model. **(C,D)** WT and Ctse^−/−^ C57Bl/6 mice were used as recipients in the LP/J→C57BL/6 model. **(A,C)** Survival curves. **(B,D)** Cumulated clinical score (posture, movement, fur, skin, weight; single score: 0–2, max. score: 10). Error bars indicate mean ± SEM, *p*-values in **(A,C)** were calculated using a log-rank (Mantel–Cox) test, ***p < 0.001.

To investigate the role of Ctse in regulating GVHD, we first investigated the effect of Ctse deficiency of allo-SCT donors on the development of GVHD. We used the haploidentical allo-SCT model C57BL/6 (H2^b^)→B6D2F1 (H2^b/d^). After chemotherapy with busulfan and cyclophosphamide, recipients received T cell-depleted BM (TCD-BM) and GVHD was induced by the addition of donor splenic T cells to the allograft. We used *Ctse*^−/−^ donors and WT littermate donors as the source for TCD-BM or T cells. We did not detect significant differences in lethal GVHD between the two different groups (Figure 2A). Furthermore, we did not detect differences in clinical GVHD scores in allo-SCT recipients between the *Ctse*^−/−^ donor group and WT donor group (Figure 2B).

### Ctse^−/−^ allo-SCT Recipients Have Reduced Lethal Acute GVHD

**Figure 3:**
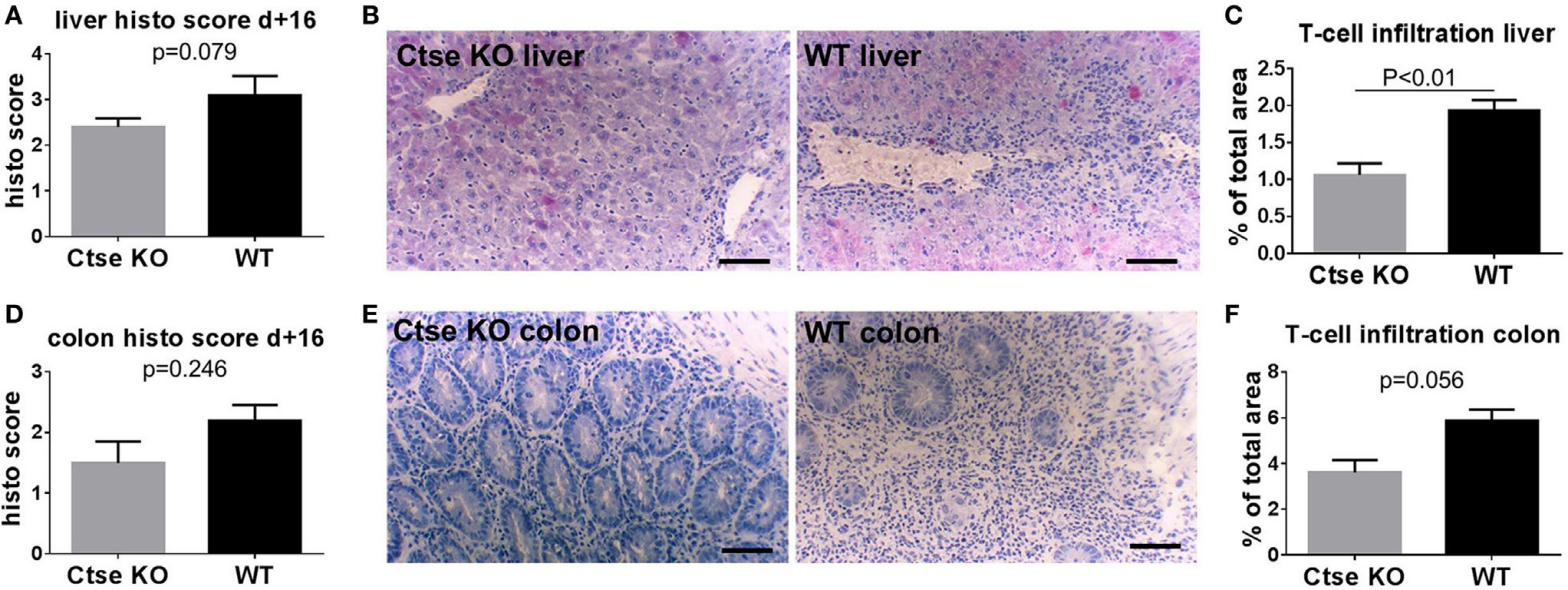
**Histopathological scores and T cell infiltration is reduced in cathepsin E (Ctse)^−/−^ allogeneic hematopoietic stem cell transplantation (allo-SCT) recipients**. **(A,D)** Score of histopathological analysis of liver and colon sections at day +16 after allo-SCT in wild-type (WT) vs. Ctse^−/−^ allo-SCT recipients. **(B,E)** Examples of H + E stainings of liver and colon sections from Ctse^−/−^ vs. WT allo-SCT recipients. **(C,F)** Analysis of T cell infiltration into liver and colon at day +16 after allo-SCT, measured by labeled lymphocyte immunofluorescence area of total liver area or total colon mucosal area in WT and Ctse^−/−^ allo-SCT recipients at day +16. *n* = 5 for all experiments. For immunofluorescence T cell infiltration analysis, six pictures per animal were taken. Bright-field images were taken using a Motic BA410 microscope with a Moticam Pro 285B and the Motic Images Plus 2.0 software. The used objective was a Plan Fluar 20×/0.50. Error bars indicate mean ± SEM, *p*-values were calculated using Wilcoxon–Mann–Whitney rank sum test. Bar = 100 μm.

Next, we used the LP/J (H2^b^)→C57BL/6 (H2^b^) model to investigate Ctse deficiency in allo-SCT recipients (26). We found significantly less lethal GVHD in B6 Ctse^−/−^ allo-SCT recipients as compared to B6 WT littermate allo-SCT recipients (Figure 2C). We performed investigator-blinded scoring for clinical GVHD symptoms and found significantly reduced clinical GVHD scores in *Ctse*^−/−^ allo-SCT recipients vs. WT littermate allo-SCT recipients (Figure 2D). To analyze target organ GVHD, we performed histopathological analyses and found less GVHD in the liver and a trend toward less GVHD in colon (Figures 3A,B,D,E). The numbers of tissue-infiltrating alloreactive T-lymphocytes were reduced in *Ctse*^−/−^ allo-SCT recipients in GVHD target organs colon and liver (Figures 3C,F) at peak GVHD development on day +16 following BMT, demonstrating less GVHD-related target organ inflammation.

### Analyses of Systemic Inflammation during GVHD in Ctse-Deficient vs. WT allo-SCT Recipients

**Figure 4:**
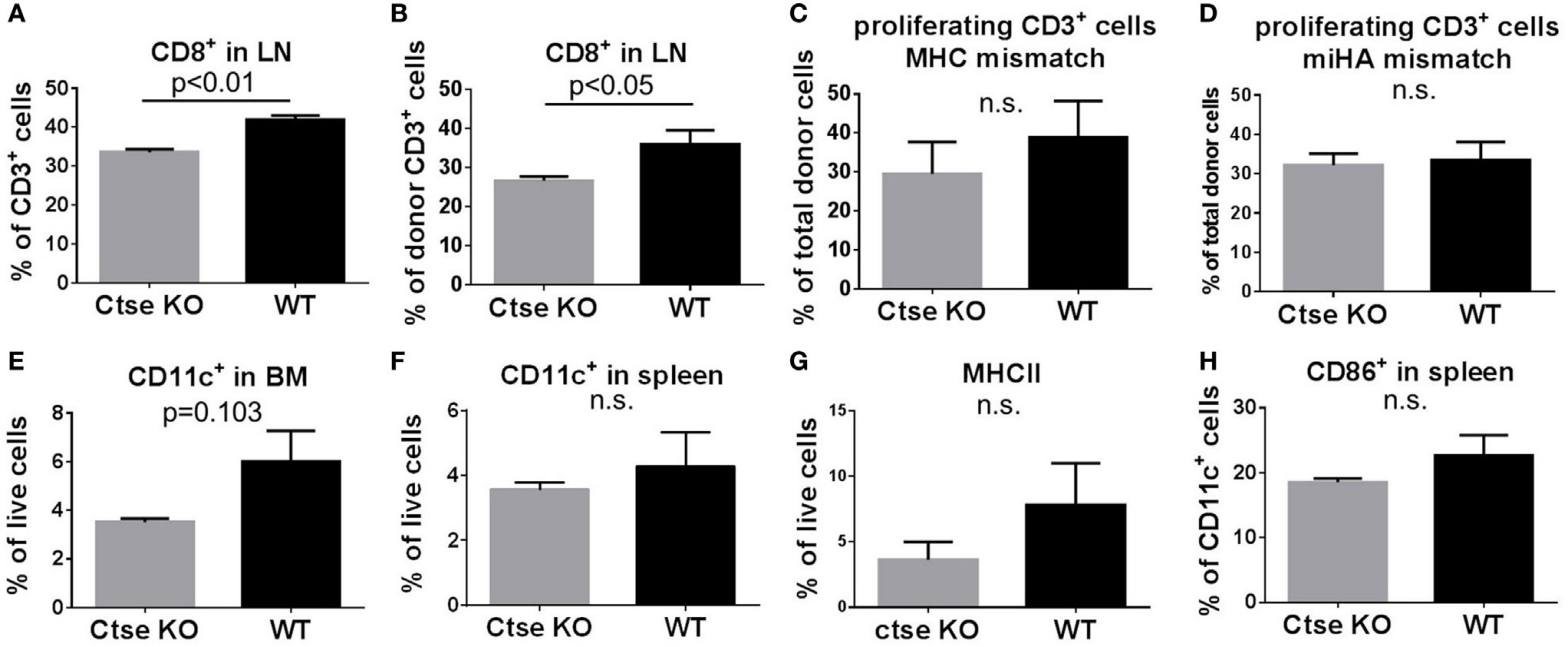
**Quantification of T cell number, T cell proliferation, and dendritic cell number in wild-type (WT) vs. cathepsin E (Ctse)^−/−^ allogeneic hematopoietic stem cell transplantation (allo-SCT) recipients**. WT vs. Ctse^−/−^ C57Bl/6 mice were used as SCT recipients. **(A,B)** Quantification of CD8^+^ cells in lymph nodes of Ctse^−/−^ and WT recipients on day +16 after allo-SCT in the LP/J→C57BL/6 model. **(C,D)** *In vivo* proliferation assay with CFSE labeled Balb/C MHC-mismatched **(C)** or 129J miHA-mismatched **(D)** CD3^+^ lymphocytes. The percentage of proliferating CD3^+^ donor T cells is shown. **(E–H)** Analyses during established graft-versus-host disease at day +16 after allo-SCT in the LP/J→C57BL/6 model. **(E,F)** Quantification of CD11c^+^ cells in spleen and bone marrow from Ctse^−/−^ vs. WT allo-SCT recipients. **(G)** Quantification of MHC II^+^ host cells in the spleen of Ctse^−/−^ vs. WT allo-SCT recipients. **(H)** Quantification of CD86^+^ host cells in the spleen of Ctse^−/−^ vs. WT allo-SCT recipients. Data were obtained with applying the markers of interest to the live cell gate. *n* = 5 animals per group and per experiment. Error bars indicate mean ± SEM, *p*-values were calculated using Wilcoxon–Mann–Whitney rank sum test.

To analyze the impact of Ctse deficiency on engraftment and on systemic inflammation during GVHD, we sacrificed Ctse^−/−^ allo-SCT recipients and WT allo-SCT recipients in the LP/J (H2^b^)→C57BL/6 (H2^b^) model at day +16 after allo-SCT. We harvested blood, BM, thymus, spleen, and peripheral lymph nodes for flow cytometric analyses. In these experiments, we found similar numbers for donor chimerism and for most lymphoid and myeloid cell populations in the blood, BM, thymus, spleen, and peripheral lymph nodes of Ctse-deficient vs. WT allo-SCT recipients (Figures S1–3 in Supplementary Material). Of note, we found no significant differences of donor T cell numbers in blood and spleen (Figures S2 and S3 in Supplementary Material). However, in lymph nodes, we found significantly reduced CD8^+^ T cell counts in Ctse-deficient allo-SCT recipients vs. WT allo-SCT recipients (Figures 4A,B). The number of donor CD8^+^ T cells were reduced (Figure 4B), leading to an overall reduction of total CD8^+^ T cells (Figure 4A). Due to these results and because of the previously described role of Ctse on antigen presentation, we wanted to determine the impact of Ctse deficiency on proliferation of allogeneic donor T cells. Ctse KO C57BL/6 mice and WT littermates were conditioned with chemotherapy and CFSE-labeled, miHA-mismatched CD3^+^ lymphocytes from 129J donors or CD3^+^ lymphocytes from Balb/c (major MHC mismatch) were injected i.v. into the tail vein. Ninety-six hours later, cells were isolated from spleen and lymph nodes for flow cytometric analyses. The proportion of proliferating T cells was not significantly different between Ctse-deficient recipients vs. WT recipients in both models, neither in percent of less CFSE-labeled T cells (counted as proliferating cells) compared to control CFSE labeled T cells (as shown, Figures 4C,D; Figures S5 and S6 in Supplementary Material) nor in the mean fluorescence index of CFSE positive cells in these assays (calculated by FlowJo software, data not shown). Furthermore, we found that Ctse deficiency of DCs had no significant impact on the *in vitro* proliferation rate of allogeneic T cells in MLRs (Figure S4 in Supplementary Material). Taken together, we found in our model and assays no major impact of Ctse on the ability of DCs to induce alloactivation of donor T cells.

To further investigate the impact of Ctse deficiency of the allo-SCT recipient on host DCs, we analyzed DC numbers and activation status during GVHD in BM, lymph nodes, and spleen. We found no significant differences in DC counts between Ctse-deficient allo-SCT recipients vs. WT allo-SCT recipients in lymph nodes. However, there was a non-significant trend toward reduced CD11c^+^ DC numbers in BM and spleen (Figures 4E,F) as well as a trend toward lower MHCII expression on live cells and CD86 expression of Ctse-deficient DCs during GVHD (Figures 4G,H). Other activation markers, like CD40 and CD80, were found to be expressed similar in Ctse-deficient vs. WT animals at day +16 post BMT (data not shown). Of note, at the day of analysis (day +16 post allo-SCT) around 60–70% of all cells in the investigated lymphoid organs were donor marker Ly9.1 negative.

### The Numbers of Tissue-Infiltrating DCs Is Reduced in Livers of Ctse-Deficient allo-SCT Recipients during GVHD

**Figure 5:**
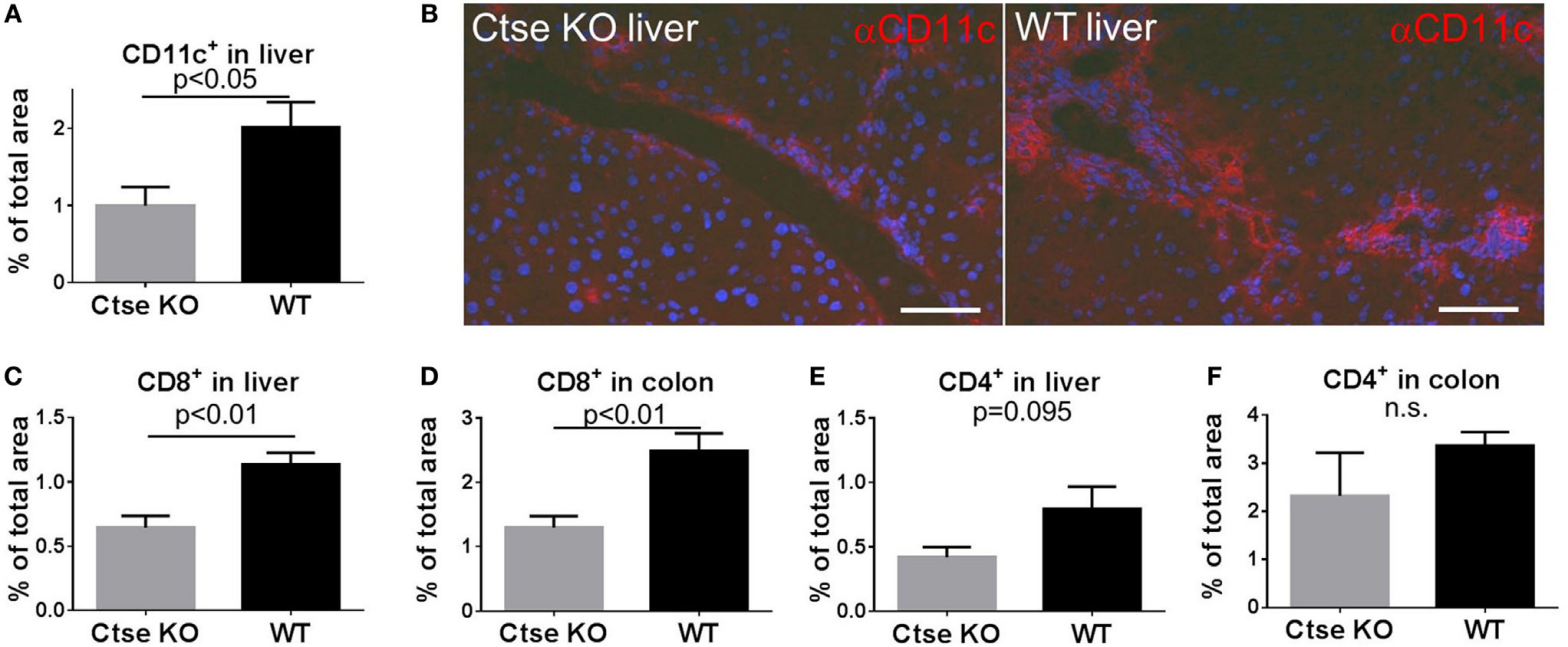
**Reduced CD11c^+^ and CD8^+^ cell counts in graft-versus-host disease target organs of cathepsin E (Ctse)-deficient allogeneic hematopoietic stem cell transplantation (allo-SCT) recipients**. **(A,B)** Analysis of CD11c-expressing cells in cryosections of Ctse^−/−^ vs. wild-type(WT) allo-SCT recipients on day +16 after SCT in the LP/J→C57BL/6 model. **(B)** Representative images of CD11c IF labeling in liver of Ctse^−/−^ vs. WT allo-SCT recipients. **(C–F)** Analysis of CD4^+^ or CD8^+^ T cell infiltration into colon and liver of Ctse^−/−^ vs. WT allo-SCT recipients, measured by labeled lymphocyte immunofluorescence area of total liver area or total colon mucosal area. *n* = 5 animals per group and per experiment. Fluorescence images were taken using a Motic BA410 microscope with a Moticam Pro 285B and the Motic Images Plus 2.0 software. The used objective was a Plan Fluar 40×/0.75. Error bars indicate mean ± SEM, *p*-values were calculated using Wilcoxon–Mann–Whitney rank sum test. Bar = 50 μm.

Next, we were interested in tissue-infiltrating DCs during GVHD. For analyses, we chose liver and omitted colon because in colon CD11c expression is found on a substantial proportion of macrophages, which is a confounder (28). We used the LP/J (H2^b^)→C57BL/6 (H2^b^) model and found significantly less liver-infiltrating CD11c^+^ cells in Ctse-deficient allo-SCT recipients vs. WT allo-SCT recipients (Figures 5A,B). In addition, we found reduced numbers of tissue-infiltrating CD8^+^ T cells in liver and colon of Ctse-deficient allo-SCT recipients during GVHD (Figures 5C,D). Again, CD4^+^ T cell infiltration into liver and colon was found to be only slightly reduced and was not statistically significant (Figures 5E,F).

### The Motility of Ctse-Deficient DCs Is Impaired

In our experiments, the reduced DC counts in liver in *Ctse*^−/−^ allo-SCT recipients during GVHD were striking. Importantly, overexpression of Ctse has been shown by different groups to increase the invasive and migratory capacity of cancer cells (24, 29, 30), raising the possibility that Ctse is a positive regulator of cell motility. Additionally, it was shown by Chen et al. (31) and Reichardt et al. (32) that DC motility influences GVHD development. We therefore hypothesized that Ctse deficiency does have an influence on DC motility, thus leading to reduced migration of DCs to target organs and a lower degree of tissue inflammation in Ctse-deficient allo-SCT recipients during GVHD.

**Figure 6:**
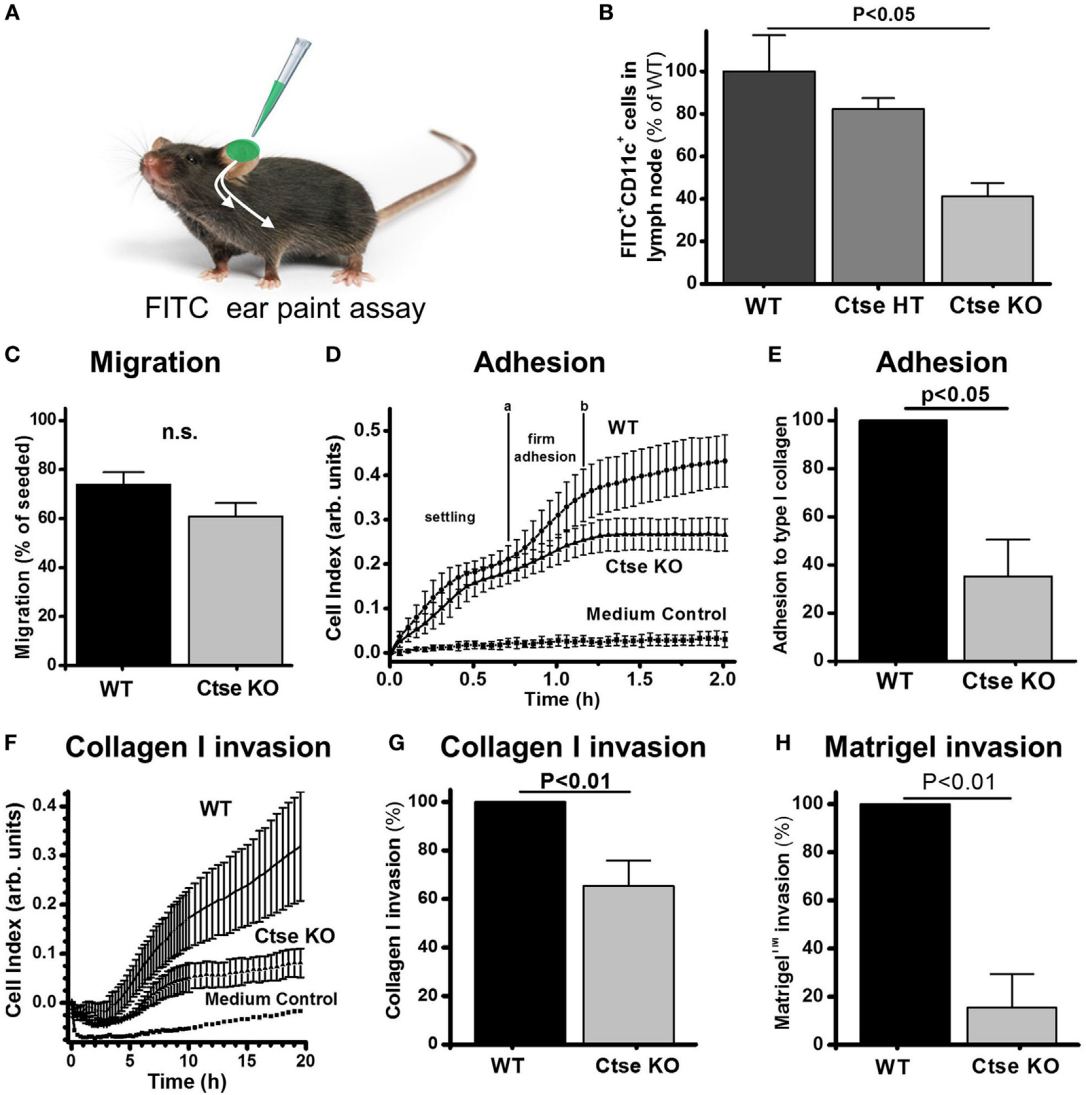
**Cathepsin E (Ctse)-deficient dendritic cells (DCs) show impaired adhesion and invasion potential**. **(A)** Experimental setup of determination of FITC-labeled DC (FITC^+^ CD11c^+^) migration to draining lymph nodes. Measurement was done 24 h after local application of irritant-solved FITC to the ear skin. **(B)** Quantification of FITC^+^ CD11c^+^ cells in the lymph nodes of Ctse^+/+^ (*n* = 11), Ctse^+/−^ (*n* = 3), and Ctse^−/−^ (*n* = 11) mice. **(C)** DC migration through a porous uncoated membrane in a Boyden Chamber (*n* = 3). **(D–H)** Adhesion and invasion was quantified by impedance measurements using the xCelligence system (ACEA) and plotted as Cell Index. **(D)** Representative adhesion measurement on collagen I coated surface. The slope between the time points a and b, where firm adhesion occurs, was further analyzed. **(E)** Quantification of DC adhesion on collagen I (*n* = 3). **(F)** Representative invasion assay through a layer of collagen I in presence of LPS in the upper well and CCL19 and CCL21 in the lower well. **(G)** Quantification of DC invasion through collagen I (1.73 mg/ml; *n* = 3), and **(H)** Matrigel™ (9 mg/ml; *n* = 3). Error bars indicate mean ± SD, *p*-values were calculated using non-parametric Kruskal–Wallis ANOVA and a *post hoc* Mann–Whitney *U*-test **(B)**, the two-sided two sample *t*-test **(C)** or two-sided one-sample *t*-test for the normalized data in panels **(E,G,H)**.

Following our hypothesis, we analyzed DCs from *Ctse*^−/−^ and WT animals for important motility characteristics of APCs. In an FITC ear paint assay (Figure 6A), we found a 60% reduction in FITC^+^ CD11c^+^ cell counts in the draining lymph nodes of *Ctse*^−/−^ mice as compared to WT mice (Figure 6B). Interestingly, we (Figure 6C) and others could not show any significant difference in transwell migration of Ctse-deficient DCs compared to WT DCs following a chemoattractant stimulus (11). The movement of cells *in vivo* is, however, not solely dependent on their intrinsic motility but also depends on the ability to form contacts with the extracellular matrix (ECM) and to actively transmigrate through matrix-rich areas. While adhesion to ECM components is one important step in entering, for example, vascular structures, to migrate from the periphery to distant locations, like lymph nodes and other tissues *in vivo*, the second crucial step is transmigration through these ECM structures like the basal membrane of endo- and epithelial layers. Here, we could show that both steps in this process, the adhesion to (Figures 6D,E) and the transmigration through ECM substrates like collagen and matrigel (Figures 6F–H), were markedly reduced between 40 and 80% in *in vitro* assays using Ctse-deficient DCs compared to WT DCs. We conclude that Ctse is involved in regulating the motility of DCs.

### Analysis of the Impact of Ctse Deficiency on Tumor Growth

**Figure 7:**
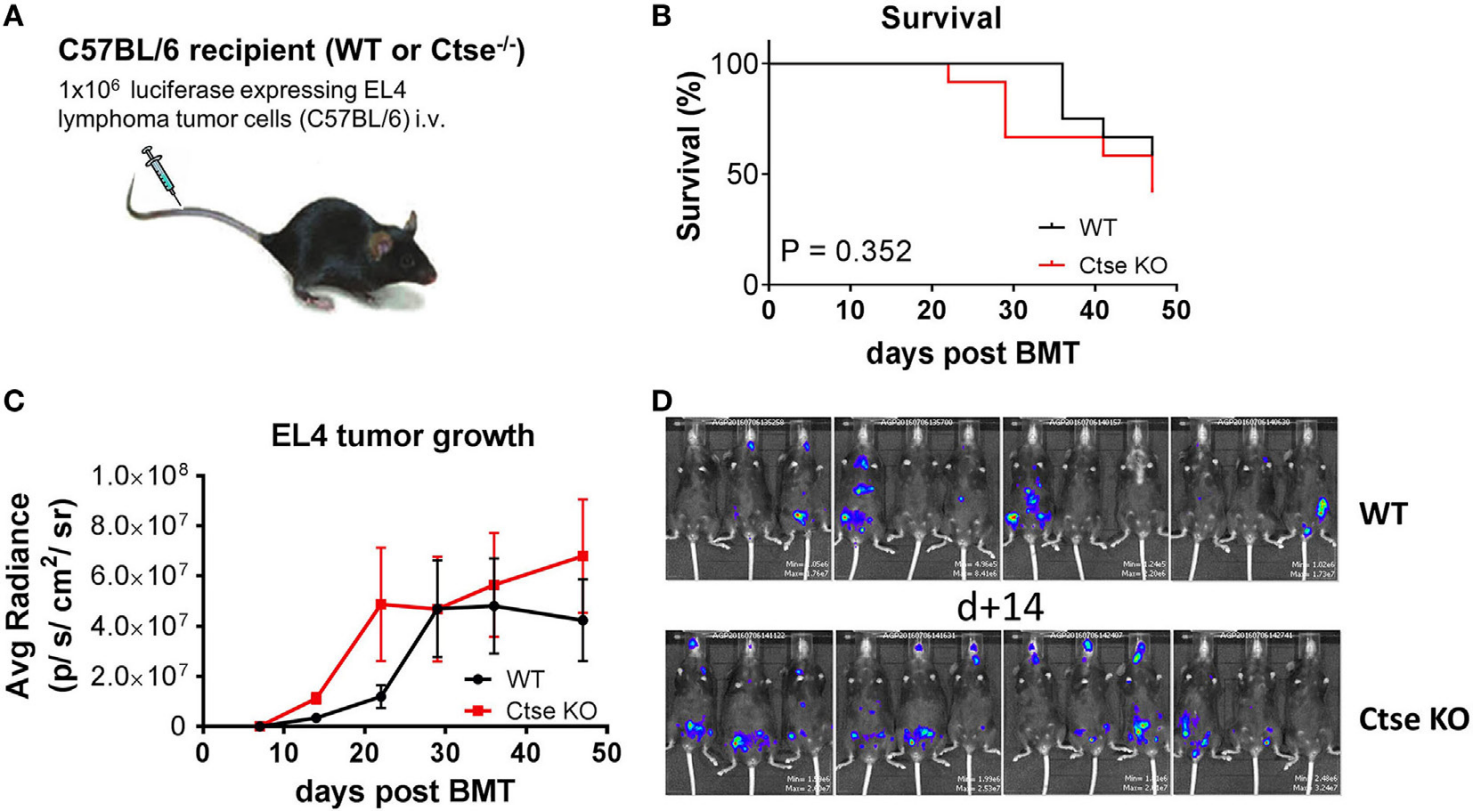
**Analysis of EL4 T cell lymphoma growth in untreated cathepsin E (Ctse)-deficient mice vs. wild-type (WT) mice**. C57Bl/6 WT or Ctse KO mice were injected intravenously with 1 × 10^6^ luciferase-expressing EL4 tumor cells. **(A)** Schematic representation of the tumor model. **(B)** Survival curve of WT and Ctse^−/−^ (Ctse KO) mice after EL4 lymphoma cell injection, statistical analysis was done using the Mantel–Cox log-rank test. **(C)** Average radiance data of WT and Ctse^−/−^ mice after tumor cell injection. *n* = 12 per group. Error bars indicate mean ± SEM. Day 14 after tumor challenge *p* < 0.01; all other time points not significant. **(D)** Pictures of WT and Ctse^−/−^ mice on day 14 after EL4 T cell lymphoma injection.

A possible role of Ctse in antitumor immunity has been suggested by others (33, 34). We therefore studied the role of Ctse in antitumor responses in C57BL/6 WT mice vs. C57BL/6 *Ctse*^−/−^ mice (Figure 7A). Mice were challenged intravenously with EL4 T cell lymphoma cells. We found no significant differences in tumor-associated mortality and survival (Figure 7B). In bioluminescence imaging, we found no significant differences of tumor growth in *Ctse*^−/−^ mice vs. WT mice at most time points (Figure 7C). However, at the earliest time point that we chose for imaging (day 14 after tumor challenge), tumor load was moderately, but significantly, higher in *Ctse*^−/−^ mice vs. WT mice (Figure 7D). Taken together, our data argue against a major impact of Ctse on immunity against EL4 lymphoma.

## Discussion

We found upregulation of Ctse in target organs during GVHD that was mainly caused by tissue infiltration of Ctse-expressing immune cells, such as CD11c^+^ cells. Our data, positively, connect to previous reports on increased Ctse activity in inflammatory and infectious diseases (14, 15). The restriction of Ctse expression to immune cells is in line with previous publications showing that Ctse is predominantly expressed in antigen-presenting cells (6, 8). Furthermore, it was shown that Ctse was not expressed in T cells and we found its deficiency on the donor side had no significant influence on GVHD, suggesting that Ctse has no important direct impact on alloactivation of T cells.

Using *Ctse*^−/−^ and WT littermate transgenic mice, we found that Ctse deficiency in allo-SCT recipients ameliorates acute GVHD. In histology, the effect of Ctse deficiency on target organ histology was stronger in liver, as compared with colon (Figure 3). In contrast, when we used *Ctse*^−/−^ and WT littermate transgenic mice as allo-SCT donors, we found no significant impact on GVHD. These results demonstrate that the Ctse deficiency on the recipient side is involved in GVHD regulation. Our data provide novel evidence on the significant biologic role of Ctse in alloreactivity and inflammation. This information adds to the existing evidence on the important role of molecules that are involved in sensing and processing of bacterial peptides, such as nod-like-receptors and toll like receptors (2). To our knowledge, this is the first report on the role of Ctse during allo-SCT. One previous report associated Cathepsin S (Ctss), a lysosomal cysteine protease, with the development of GVHD. *Ctss* mRNA expression and activity was upregulated after allo-SCT. However, the pharmacologic inhibition of Ctss led to increased GVHD, possibly because of cross presentation to MHC class I (35). Our data on Ctse during GVHD are in line with our previous publication on reduced inflammation in asthma models in *Ctse*^−/−^ mice (25). However, our findings are in some disagreement to *in vitro* investigations with an independently generated *Ctse*^−/−^ mouse showing that Ctse deletion reduced OVA-stimulated T cell activation by *Ctse*^−/−^ macrophages but enhanced T cell response by *Ctse*^−/−^ DCs (11). These discrepancies might be due to variable cell type- and antigen-specific roles of proteases in antigen presentation, and also highlights the importance of systematic and careful dissection of the role of Ctse for DC function in complex *in vivo* systems such as our GVHD model.

To investigate the mechanism how Ctse influences GVHD, we were first interested in the ability of antigen-presenting cells, such as DCs, to induce allogeneic T cell proliferation. In MHC-matched and MHC-mismatched models, we found that Ctse deficiency in antigen-presenting cells had no major impact on alloactivation of WT donor T cells *in vivo* and *in vitro*. Although robust data exist showing that Ctse has important functions in antigen presentation, our data argue against a major biologic relevance of Ctse-mediated antigen presentation during GVHD. In search for alternative mechanisms how Ctse regulates GVHD, we became interested in DC motility because of the significantly reduced DC numbers we found during hepatic GVHD in Ctse-deficient allo-SCT recipients. We demonstrated that Ctse deficiency results in profoundly impaired DC motility and adhesion to and migration through ECM. These results might be the explanation for the reduced DC numbers in GVHD target organs of Ctse-deficient mice compared to WT animals. To our knowledge, this is the first report on the impact of cathepsin proteases during DC motility. Of note, we found no significant differences in DC numbers in lymph nodes of Ctse-deficient allo-SCT recipients vs. WT allo-SCT recipients. Based on previous knowledge and our experimental data, we are currently unable to provide a mechanistic explanation of this discrepancy between DC numbers lymph nodes and the liver. Another limitation of our study is, that we were unable to prove, that the observed effects are mediated by Ctse activity in DCs. It is also possible that Ctse-mediated effects in other recipient cell types, e.g., in macrophages, may have contributed to the observed effects. Others (31, 32) have shown that either miR-155 deficiency in the recipient DC compartment or rapamycin-treated host DCs led to impaired GVHD development. In both studies, the motility of DCs was shown to be affected as well as the expression of surface receptors and cytokine expression in the investigated DC populations. In addition, it has previously been demonstrated that Ctse expression in cancer cells is associated with increased invasiveness, linking Ctse to cell motility (24, 29, 30). Others could show decreased DC motility in mice deficient for syndecan-4 or heparanase (36, 37). Similar to Ctse, both proteins are related to ECM interaction and degradation, and in both cases, reduced DC motility led to reduced inflammation in the respective models. Regarding clinical development, Ctse is a feasible target for small molecule inhibitors, like pepstatin A (38) or other aspartic protease inhibitors (39). Taken together, previous studies provided evidence for the biologic significance of DC motility. Our findings on reduced DC motility in Ctse-deficient mice provide a possible link between microbial products and DC motility.

## Author Contributions

JM, TR, and OP designed the study. JM, LB, YS, KR, MK, SM, and SC performed the experiments and analyzed the data. JM, TR, and OP wrote the manuscript.

## Conflict of Interest Statement

The authors declare that the research was conducted in the absence of any commercial or financial relationships that could be construed as a potential conflict of interest.
