# Supplementary material for: Cathepsin E Deficiency Ameliorates Graft-versus-Host Disease and Modifies Dendritic Cell Motility
Source: Front Immunol. 2017 Mar 1;8:203. doi: 10.3389/fimmu.2017.00203 (PMC5331043; doi:10.3389/fimmu.2017.00203)
Supplement: Supplementary file 1 [file Presentation_1.ZIP › Figures.DOCX]

Supplemental Data

Supplemental Figure 1

**
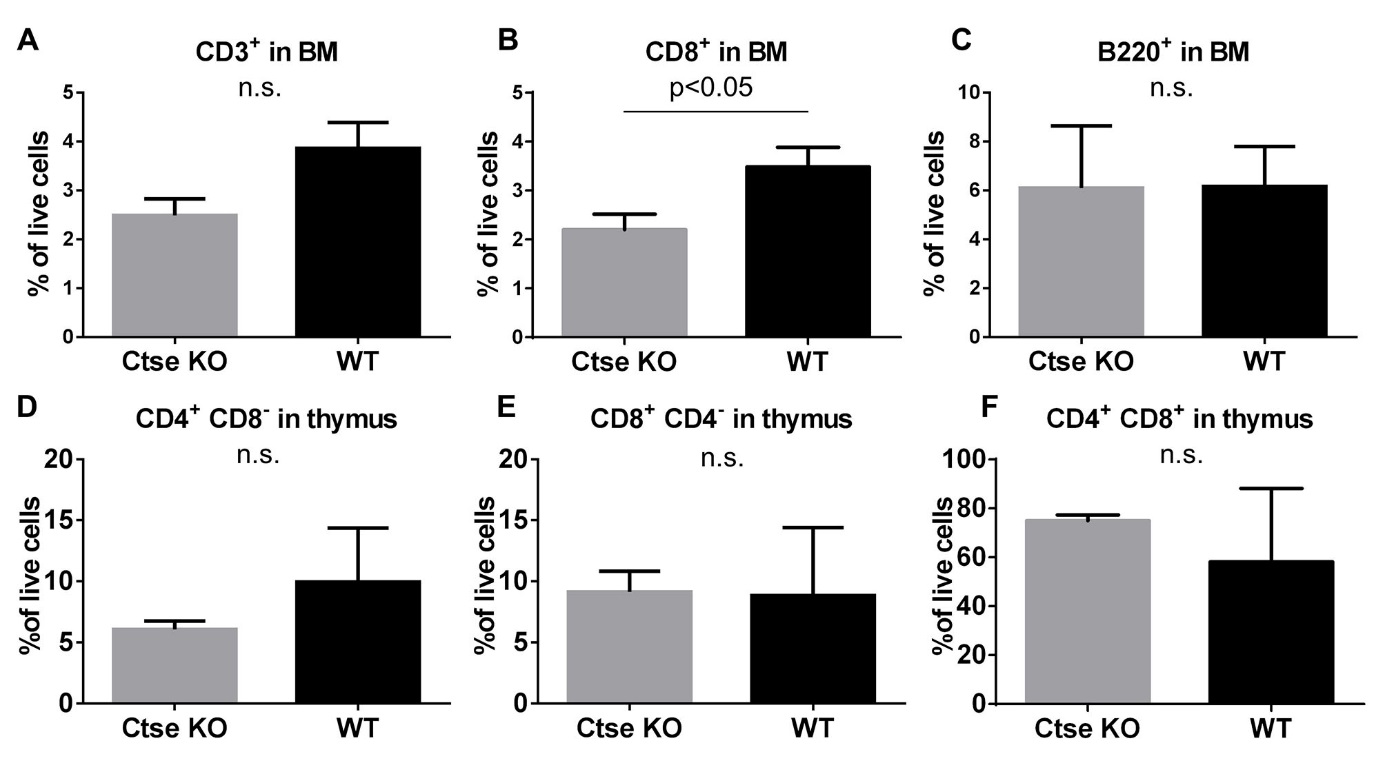
**

Supplemental Figure 2

**
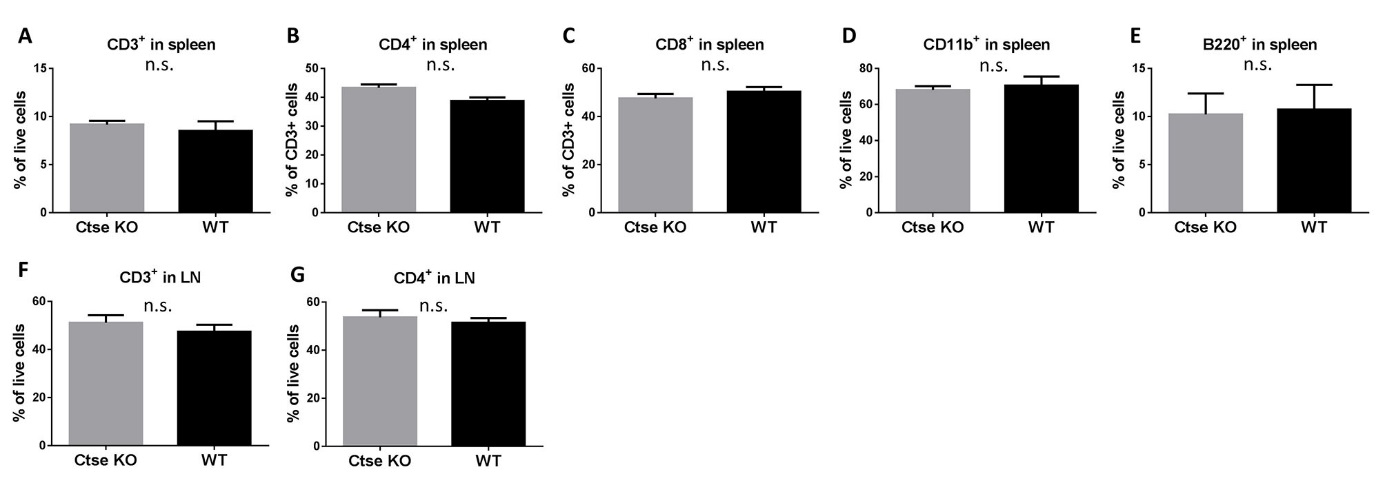
**

Supplemental Figure 3


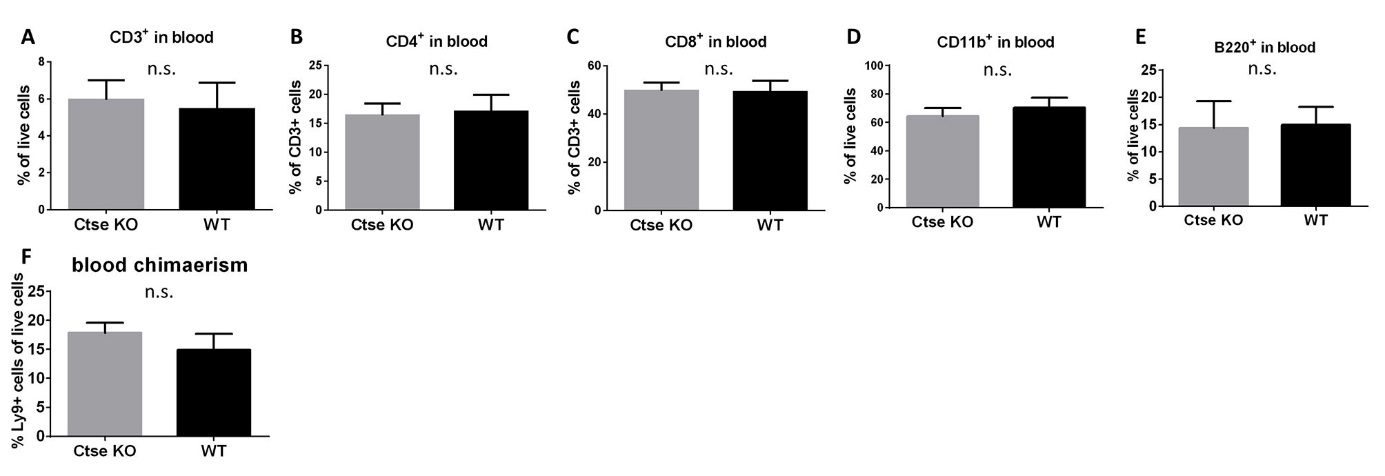


Supplemental Figure 4

**
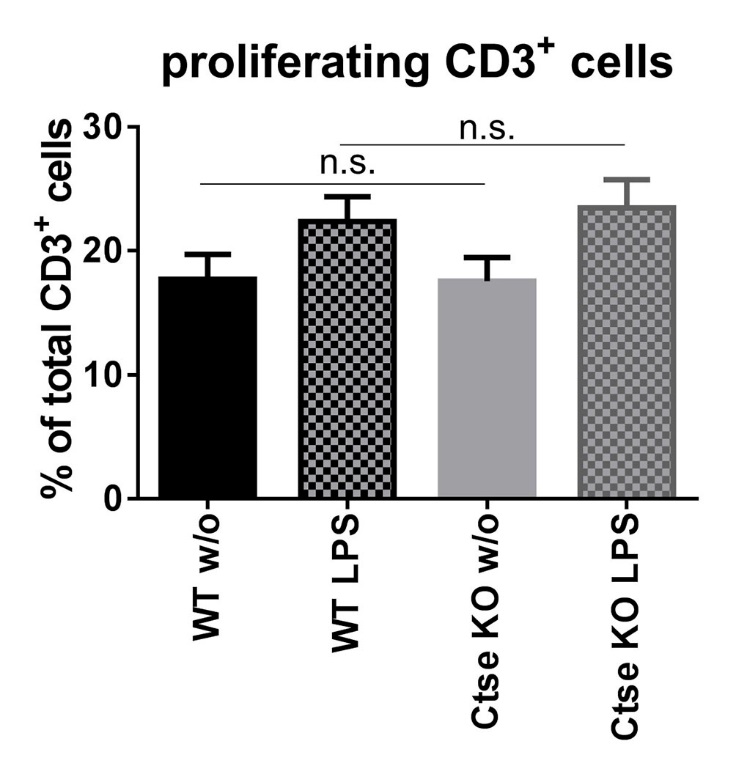
**


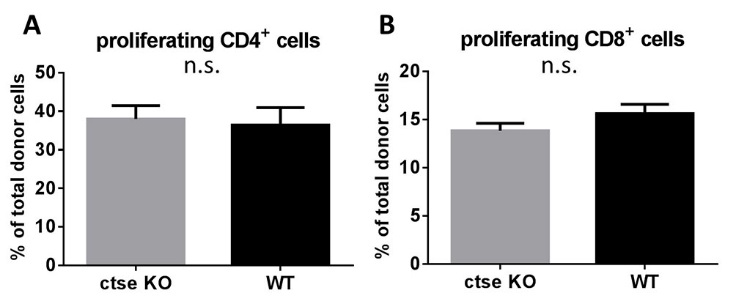
Supplemental Figure 5

Supplemental Figure 6
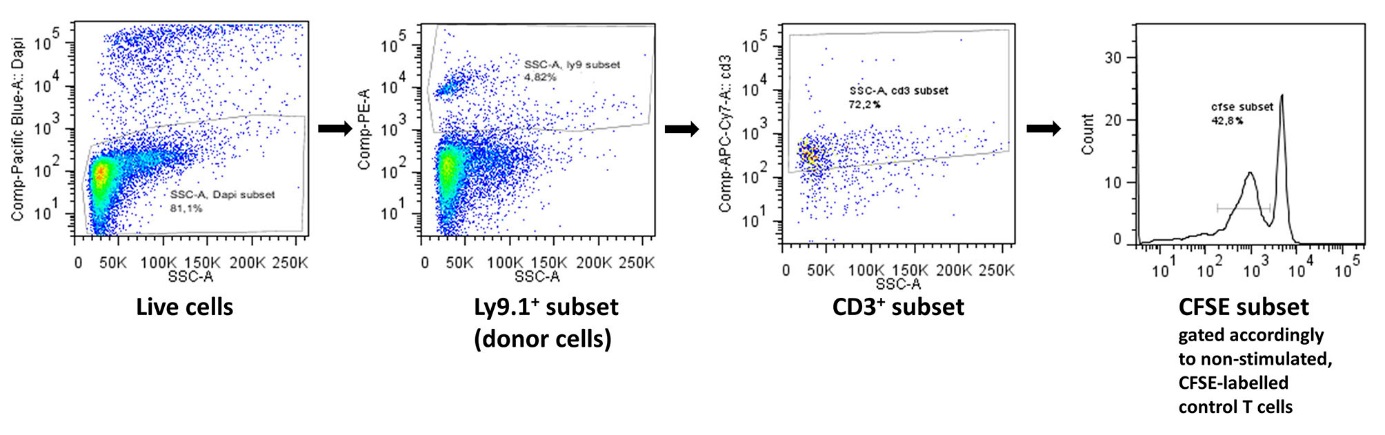


Supplemental figure 7

**
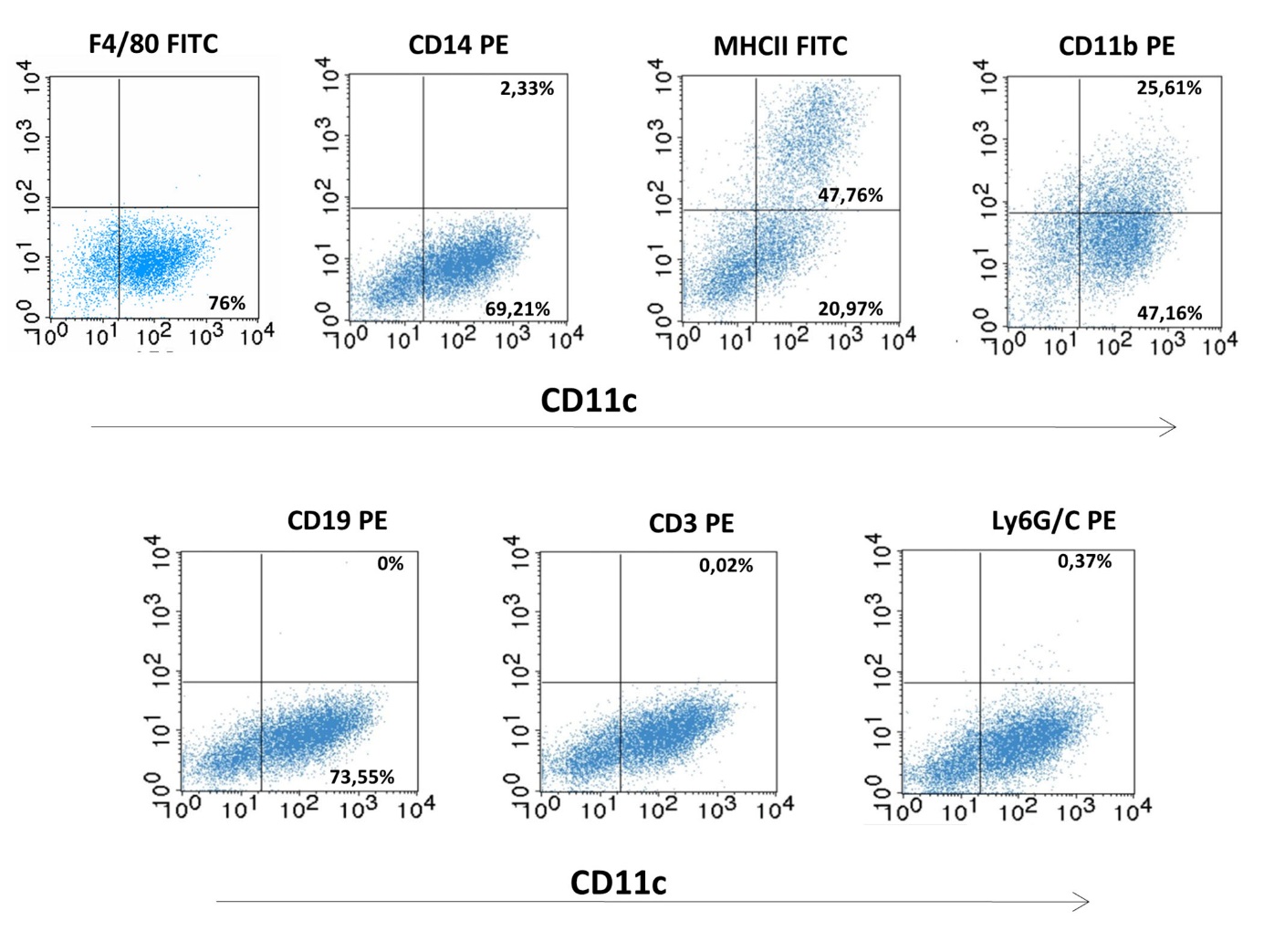
**

**Suppl. Figure 1: Lymphoid and myeloid cell populations in primary lymphoid organs during acute GVHD in Ctse^-/-^** **and WT mice.** **A-C)** FACS analysis of different lymphoid and myeloid cell populations at day+16 after BMT isolated from bone marrow and **D-F)** thymus of Ctse^-/-^ and WT allo-SCT recipients, stained for the respective marker prior to analysis. n=5 animals per group. Error bars indicate mean ± SEM, p-values were calculated using Wilcoxon-Mann-Whitney rank sum test.

**Suppl. Figure 2: Lymphoid and myeloid cell populations in secondary lymphoid organs during acute GVHD in Ctse^-/-^** **and WT mice. A-E)** FACS analysis of different lymphoid and myeloid cell populations at day +16 after BMT isolated from spleen and **F+G)** lymph nodes of Ctse^-/-^ and WT allo-SCT recipients, stained for the respective marker prior to analysis. n=5 animals per group. Error bars indicate mean ± SEM, p-values were calculated using Wilcoxon-Mann-Whitney rank sum test.

**Suppl. Figure 3: Donor chimaerism and lymphoid and myeloid cell populations in blood during acute GVHD in Ctse^-/-^** **and WT mice. A-E)** FACS analysis of different lymphoid and myeloid cell populations at day +16 after BMT isolated from blood of Ctse^-/-^ and WT allo-SCT recipients, stained for the respective marker prior to analysis. **F)** Facs analysis of blood donor chimaerism in Ctse-/- and WT allo-SCT recipients at day +16 after BMT with Ly9.1 donor cell marker. n=5 animals per group. Error bars indicate mean ± SEM, p-values were calculated using Wilcoxon-Mann-Whitney rank sum test.

**Suppl. Figure 4: No significant differences in the ability to induce allogeneic T cell proliferation of Ctse deficient DCs vs WT DCs in *in vitro* MLR assays.** Shown are mixed lymphocyte reactions (MLR) with CFSE-labelled CD3^+^ cells from Balb/C mice as responder cells and either WT or Ctse^-/-^ (Ctse KO) purified CD11c^+^ cells as activators. DCs from Ctse^-/-^ (Ctse KO) and WT mice were isolated from spleens and either not treated or pre-treated with LPS at 100ng/ml for 4h. FACS analysis was performed 96h after induction. CFSE labelled CD3^+^ cells are shown in relation to total CD3^+^ cells. All CFSE positive cells that underwent at least one division were considered as proliferating cells. n=3 animals/group, two experiments, p-values were calculated using a double-sided student‘s T-test.

**Suppl. Figure 5: No significant differences in the ability to induce allogeneic CD4^+^ or CD8^+^ T cell proliferation of Ctse deficient mice *vs* WT mice in *in vivo* proliferation assays.** Shown are results from *in vivo* proliferation assays with CFSE-labelled CD3^+^ cells from LP/J mice as responder cells injected into either Ctse deficient mice or WT littermates. FACS analysis was performed 96h after induction. CFSE labelled CD4^+^ (A) or CD8^+^ (B) cells are shown in relation to total donor cells respectively. All CFSE positive cells that underwent at least one division were considered as proliferating cells. n=4 animals/group. Error bars indicate mean ± SEM, p-values were calculated using Wilcoxon-Mann-Whitney rank sum test.

**Suppl. Figure 6: Gating strategy for in vivo proliferation assays.** Shown is our gating strategy used for the analysis of the in vivo proliferation assays. DAPI labeling was used to discriminate dead from live cells, followed by the Ly9.1^+^ gate to identify donor cells. As the next gate CD3^+^ cells were separated and finally in this cell population the CFSE gate was drawn accordingly to non-stimulated, CFSE-labeled CD3^+^ T cells to count only proliferating cells (that show lower CFSE labeling than the non-proliferating cells).

**Suppl. Figure 7: Phenotype of bone marrow derived dendritic cells from *Ctse^+/+^* mice.** Flow cytometric analysis of dendritic cells stained with anti-CD11c-APC antibody in combination with the indicated markers at day 7 of cell culture.

Supplemental methods:

*Mixed leukocyte reaction*

# DCs were isolated from spleen of C57BL/6 ctse-/- and WT mice using a CD11c+ isolation kit and splenic T cells from LP/J mice were obtained using the mouse Pan T cell isolation Kit II (Miltenyi Biotec, Bergisch Gladbach, Germany) according to the manufactors instructions. DCs were treated with 100ng/ml LPS or left untreated for 4 h. T-cells were loaded with CFSE (5-(and-6)-Carboxyfluorescein Diacetate, Succinimidyl Ester, Thermo Fisher). 2.5x10^4^ DCs (activators) and 2.5x10^5^ T-cell (responders) were put together for 96h in an incubator at 37°C and 5% CO_2_. FACS analysis was done measuring total cell counts positive for CFSE and CD3. Proliferating cells are determined as cells, showing less CFSE load compared to control samples with only CFSE loaded T-cells.
