# Supplementary material for: Cathepsin E Deficiency Ameliorates Graft-versus-Host Disease and Modifies Dendritic Cell Motility
Source: Front Immunol. 2017 Mar 1;8:203. doi: 10.3389/fimmu.2017.00203 (PMC5331043; doi:10.3389/fimmu.2017.00203)
Supplement: Supplementary file 1 [file Presentation_1.ZIP › Table 1.PDF]

Kruskal-Wallis ANOVA (26.01.2017 14:41:20)

Notes

X-Function	Kruskal-Wallis ANOVA
User Name	reinheckel
Time	26.01.2017 14:41:20

Input Data

	Data	Range
WT	[Data3]Sheet1!WT	[1*:13*]
CTSEhet	[Data3]Sheet1!CTSEhet	[1*:3*]
CTSEko	[Data3]Sheet1!CTSEko	[1*:13*]

Descriptive Statistics

	N	Min	Q1	Median	Q3	Max
WT	11	10.8639	59.41	102.43103	151.49	187.79022
CTSEhet	3	74.26	74.26	80.2	92.08	92.08
CTSEko	11	10.92598	23.97219	34.14368	61.02274	77.23

Ranks

	N	Mean Rank	Sum Rank
WT	11	16.81818	185
CTSEhet	3	17	51
CTSEko	11	8.09091	89

Test Statistics

	Chi-Square	DF	Prob>Chi-Square
	8.7407	2	0.01265

Null Hypothesis:The samples come from the same population
Alternative Hypothesis:The samples come from different populations
: At the 0.05 level, the populations are significantly different
