# Supplementary material for: Cathepsin E Deficiency Ameliorates Graft-versus-Host Disease and Modifies Dendritic Cell Motility
Source: Front Immunol. 2017 Mar 1;8:203. doi: 10.3389/fimmu.2017.00203 (PMC5331043; doi:10.3389/fimmu.2017.00203)
Supplement: Supplementary file 1 [file Presentation_1.ZIP › Table 2.PDF]

Mann-Whitney Test (26.01.2017 14:43:26)

Notes

X-Function	Mann-Whitney Test
User Name	reinheckel
Time	26.01.2017 14:43:26

Input Data

	Data	Range
1st Data Range	[Data3]Sheet1!WT	[1*:13*]
2nd Data Range	[Data3]Sheet1!CTSEko	[1*:13*]

Descriptive Statistics

	N	Min	Q1	Median	Q3	Max
WT	11	10.8639	59.41	102.43103	151.49	187.79022
CTSEko	11	10.92598	23.97219	34.14368	61.02274	77.23

Ranks

	N	Mean Rank	Sum Rank
WT	11	15	165
CTSEko	11	8	88

Test Statistics

	U	Z	Prob>|U|
	99	2.49527	0.01259

Null Hypothesis: F(x) = G(y)
Alternative Hypothesis: F(x) <> G(y)
At the 0.05 level, the two distributions are significantly different.
