# Supplementary material for: Cathepsin E Deficiency Ameliorates Graft-versus-Host Disease and Modifies Dendritic Cell Motility
Source: Front Immunol. 2017 Mar 1;8:203. doi: 10.3389/fimmu.2017.00203 (PMC5331043; doi:10.3389/fimmu.2017.00203)
Supplement: Supplementary file 1 [file Presentation_1.ZIP › Table 4.PDF]

Two sample t Test (26.01.2017 17:48:12)

Notes

X-Function	Two sample t Test
User Name	reinheckel
Time	26.01.2017 17:48:12

Input Data

	Data	Range
1st Data Range	[Data1]Sheet1!WT	[1*:3*]
2nd Data Range	[Data1]Sheet1!CTSEko	[1*:3*]

Descriptive Statistics

		N	Mean	SD	SEM
WT		3	73.8	8.87778	5.12559
CTSEko		3	60.81667	9.49455	5.48168
	Difference		12.98333		

t-Test Statistics

	t Statistic	DF	Prob>|t|
Equal Variance Assumed	1.73003	4	0.15868
Equal Variance NOT Assumed	1.73003	3.98209	0.159

Null Hypothesis: mean1-mean2 = 0
Alternative Hypothesis: mean1-mean2 <> 0
At the 0.05 level, the difference of the population means is NOT significantly different with the test difference(0)
