# Supplementary material for: Cathepsin E Deficiency Ameliorates Graft-versus-Host Disease and Modifies Dendritic Cell Motility
Source: Front Immunol. 2017 Mar 1;8:203. doi: 10.3389/fimmu.2017.00203 (PMC5331043; doi:10.3389/fimmu.2017.00203)
Supplement: Supplementary file 1 [file Presentation_1.ZIP › Table 6.PDF]

Normality Test (26.01.2017 16:17:32)

Notes

Description	Perform Normality Test
User Name	reinheckel
Operation Time	26.01.2017 16:17:32
Report Status	New Analysis Report

Input Data

	Data	Range
Data	[Book1]Sheet1!CTSEKO	[1*:3*]

Masked Data - Values Excluded from Computations

Notes
No Masked Data

Bad Data (missing values) -- Values that are invalid and thus not used in computations

Notes
No Missing Data

Descriptive Statistics

	N Analysis	N Missing	Mean	Standard Deviation	SE of Mean
CTSEKO	3	0	35.28567	15.84538	9.14834

NormalityTest

Shapiro-Wilk

	DF	Statistic	p-value	Decision at level(5%)
CTSEKO	3	0.95066	0.57218	Can't reject normality
