# Supplementary material for: Cathepsin E Deficiency Ameliorates Graft-versus-Host Disease and Modifies Dendritic Cell Motility
Source: Front Immunol. 2017 Mar 1;8:203. doi: 10.3389/fimmu.2017.00203 (PMC5331043; doi:10.3389/fimmu.2017.00203)
Supplement: Supplementary file 1 [file Presentation_1.ZIP › Table 8.PDF]

One Sample t Test (26.01.2017 16:36:18)

Notes

X-Function	One Sample t Test
User Name	reinheckel
Time	26.01.2017 16:36:18

Input Data

	Data	Range
CTSEko	[Book1]Sheet1!CTSEko	[1*:3*]

Descriptive Statistics

	N	Mean	SD	SEM
CTSEko	3	65.43233	10.40976	6.01008

Test Statistics

	t Statistic	DF	Prob>|t|
CTSEko	10.8871	2	0.00833

Null Hypothesis: Mean = 0
Alternative Hypothesis: Mean <> 0
CTSEko: At the 0.05 level, the population mean is significantly different with the test mean (0)

Powers

	Alpha	Sample Size	Power
CTSEko	0.05	3	0.99706
	0.05	50	1
	0.05	100	1
	0.05	200	1
