# Supplementary material for: Cathepsin E Deficiency Ameliorates Graft-versus-Host Disease and Modifies Dendritic Cell Motility
Source: Front Immunol. 2017 Mar 1;8:203. doi: 10.3389/fimmu.2017.00203 (PMC5331043; doi:10.3389/fimmu.2017.00203)
Supplement: Supplementary file 1 [file Presentation_1.ZIP › Table3.PDF]

Normality Test (26.01.2017 17:45:27)

Notes

Description	Perform Normality Test
User Name	reinheckel
Operation Time	26.01.2017 17:45:27
Report Status	New Analysis Report

Input Data

	Data	Range
Data	[Data1]Sheet1!WT	[1*:3*]
	[Data1]Sheet1!CTSEko	[1*:3*]

Masked Data - Values Excluded from Computations

Notes
No Masked Data

Bad Data (missing values) -- Values that are invalid and thus not used in computations

Notes
No Missing Data

Descriptive Statistics

	N Analysis	N Missing	Mean	Standard Deviation	SE of Mean
WT	3	0	73.8	8.87778	5.12559
CTSEko	3	0	60.81667	9.49455	5.48168

NormalityTest

Shapiro-Wilk

	DF	Statistic	p-value	Decision at level(5%)
WT	3	0.85002	0.2405	Can't reject normality
CTSEko	3	0.92874	0.4839	Can't reject normality
